# Long-Term Bone Mineral Density Changes in Kidney Transplant Recipients Treated with Denosumab: A Retrospective Study with Nonequivalent Control Group

**DOI:** 10.1007/s00223-024-01218-z

**Published:** 2024-05-10

**Authors:** Angelo Fassio, Stefano Andreola, Davide Gatti, Francesco Pollastri, Matteo Gatti, Paolo Fabbrini, Giovanni Gambaro, Pietro Manuel Ferraro, Chiara Caletti, Maurizio Rossini, Ombretta Viapiana, Riccardo Bixio, Giovanni Adami

**Affiliations:** 1 Rheumatology Unit, University of Verona, Verona, Italy; 2 Nephrology Unit, University of Verona, Verona, Italy; 3 Department of Nephrology and Dialysis, Ospedale Bassini, ASST Nord Milano-Cinisello Balsamo, Milan, Italy; 4 Rheumatology Unit, Policlinico GB Rossi, 37134 Verona, Italy

**Keywords:** Bone mineral density, Chronic kidney disease, Denosumab, Kidney transplant, Osteoporosis

## Abstract

Data on the effectiveness of denosumab on osteoporosis after kidney transplantation are limited. We investigated the long-term bone mineral density (BMD) changes in kidney transplant recipients (KTRs) treated with denosumab compared to untreated KTRs. We enrolled KTRs treated with denosumab 60 mg/6 months for 4 years. An untreated group of sex and age-matched KTRs with a 1:1 ratio was included. The primary outcome was BMD changes assessed by Dual-energy X-ray Absorptiometry over 4 years. Data on serum creatinine, alkaline phosphatase (ALP), parathyroid hormone, and 25-hydroxyvitamin D were collected. All patients received oral cholecalciferol and calcium supplementation. 23 denosumab-treated KTRs were enrolled, and 23 untreated KTRs. The median time from transplant to the start of denosumab was 4 years (range 0:24). The denosumab group showed a significant increase from baseline in BMD at the lumbar spine (LS) (9.0 ± 10.7%, *p* < 0.001), and total hip (TH) (3.8 ± 7.9%, *p* = 0.041). The untreated group showed a significant decrease at all sites (− 3.0 ± 7%, *p* = 0.041 at the LS; − 6.3 ± 9.2%, *p* = 0.003 at the TH; − 6.7 ± 9.3%, *p* = 0.003 at the FN). The between-group differences in percent BMD changes were statistically significant at all sites. Similar results were found for the respective Z-scores. The ALP serum levels significantly decreased from baseline only in the denosumab group, with a significant between-group difference (*p* = 0.032). No significant differences in serum creatinine, hypocalcaemic events or acute graft rejection rates were observed. Four years of denosumab therapy were associated with increased BMD in KTRs, while untreated KTRs showed significant BMD losses at all sites.

**Supplementary Information:**

The online version contains supplementary material available at 10.1007/s00223-024-01218-z.

## Introduction

Chronic kidney disease (CKD), a worldwide spreading public health issue, is characterized by disruption of mineral and endocrine functions leading to bone abnormalities and ultimately to a higher risk of bone fractures, morbidity, and mortality [1]. The incidence of bone fractures correlates with CKD severity, and in the last years, there is a growing body of evidence supporting, in this setting, the association between fracture incidence and bone mineral density (BMD) assessed by Dual-energy X-ray Absorptiometry (DXA) [2–4]. Indeed, the assessment of BMD through DXA became recommended in CKD patients by the Kidney Disease: Improving Global Outcomes (KDIGO) recommendations in 2017 [5].

The highest toll, equally shared between males and females, is paid by individuals undergoing dialysis and kidney transplant recipients (KTRs), the latter with even greater fracture risk, especially in the first years after the transplantation surgery [6, 7]. Bone disease in KTRs is multifactorial, resulting from pre-existing bone and mineral abnormality plus bone damage acquired after transplantation [8], and it is only partially explained by the widespread chronic glucocorticoid treatment [9].

Current recommendations for KTRs with low BMD in the first 12 months after transplant suggest starting treatment with vitamin D and analogues, antiresorptive agents, or concurrent treatment [5]. Denosumab, a monoclonal antibody inhibiting the receptor activator of nuclear factor kappa-B, has proven to be an effective pharmacological agent for the treatment of bone loss [10, 11], also in patients with CKD [12]. In addition, in recent years, emerging data have been published corroborating the efficacy and safety of denosumab in KTRs, but this is limited to the early post-transplant period. The effect on BMD was greater compared with that of bisphosphonates, but some Authors described an increased risk of non-severe urinary tract infections and episodes of hypocalcaemia [13–15]. Thus far, the precise influence of denosumab treatment on bone disease and its influence on the graft function in KTRs remains uncertain. For these reasons, we performed a Pre-post study with nonequivalent control group to investigate the long-term BMD changes over 4 years in KTRs receiving treatment with denosumab compared to a cohort of age and sex-matched untreated KTRs.

## Materials and Methods

We conducted a retrospective, study employing a non-equivalent control group design. The study was conducted at the Joint Rheumatology and Nephrology bone clinic, University of Verona (Verona, Italy). We reviewed all KTRs who, upon physician’s decision (based on the clinician’s estimation of fracture risk), started treatment with denosumab 60 mg every 6 months from January 2014 to January 2018. Patients who received continuous treatment for at least 4 years, with available baseline and 4-year follow-up DXA scans were included.

An untreated cohort was selected among the overall pool of KTRs referring to the clinic who had available baseline and 4-year follow-up DXA scans. All KTRs of the denosumab group were then individually age and sex-matched with untreated KTRs with a 1:1 ratio (± 3 years tolerance for age). In the case of two or more potential eligible matches, the closest one in terms of age was selected.

The primary outcome was the difference in BMD changes assessed by DXA over 4 years between the two groups.

Patients with previous exposure to other osteoactive agents (i.e., bisphosphonates, teriparatide), patients who were on haemodialysis or peritoneal dialysis, with active cancer, or with drugs known to affect bone metabolism (except for corticosteroids, vitamin D supplements, active vitamin D compounds or analogues and calcimimetics) were excluded. All subjects also received cholecalciferol supplementation (1000 to 2000 IU daily) and supplemental calcium (in order to achieve a total calcium intake of at least 1000 mg daily).

Data on patients’ characteristics, clinical history, and pharmacological treatment were retrieved from the medical records. Baseline and follow-up data on serum creatinine, alkaline phosphatase (ALP), 25-hydroxy vitamin D (25OHD), and parathyroid hormone (PTH) were recorded. Data on serum calcium and albumin levels at baseline, month six, month twelve, and year four were also recorded. Patients were further grouped according to baseline estimated glomerular filtration rate (eGFR), calculated using the 2009 Chronic Kidney Disease Epidemiology Collaboration (CKD-EPI) equation, as follows: stage 1, eGFR higher or equal to 90 mL/min/1.73 m^2^; stage 2, eGFR 60 to 89 mL/min/1.73 m^2^; stage 3a, eGFR 45 to 59 mL/min/1.73 m^2^; stage 3b, eGFR 30 to 44 mL/min/1.73 m^2^.

DXA scans were obtained at baseline (up to 6 months before the first denosumab administration) and approximately 4 years into the follow-up. BMD values were measured for all patients through the anteroposterior L1-L4 lumbar spine (LS), total hip (TH), and femoral neck (FN). DXA scans were performed according to standard clinical routine procedures, using the following device: GE Lunar iDXA 194 system (GE Healthcare Lunar, Madison, WI, USA). BMD values, T, and Z-scores were obtained from all three sites. The ten-year probability of fracture was estimated with the Fracture Risk Assessment Tool ® (FRAX ®).

The study was conducted within the protocol 1483CESC approved by our local Ethics Committee, in accordance with the 1964 Helsinki Declaration and its later amendments or comparable ethical standards. The clinical and research activities being reported are consistent with the Principles of the Declaration of Istanbul as outlined in the “Declaration of Istanbul on Organ Trafficking and Transplant Tourism”. Written informed consent was obtained from all participants included.

### Statistical Analysis

One-way ANOVA was used to test for baseline differences between the two groups a for normally distributed variables, Mann–Whitney *U* test for non-normally distributed variables, and the chi-square test was used to compare proportions.

One-way ANOVA for repeated measures was used to test for changes in BMD, Z-scores, ALP, PTH and 25OHD from baseline within the two groups.

One-way ANOVA was also used to analyse the percent BMD changes, and the absolute changes of the Z-scores, ALP, PTH, and 25OHD at 4 years between the two groups.

One-way ANCOVA with adjustment for baseline characteristics of interest was used to test for differences in the percentage BMD changes and absolute ALP changes at 4 years (including as covariates: baseline Z-scores, PTH and 25OHD for BMD and PTH and 25OHD for ALP).

Mixed-model ANOVA with Bonferroni correction for pairwise comparisons was used to analyse the differences in the changes of serum corrected calcium within groups, and to test the time * treatment interaction between groups. In the case of a significant omnibus interaction effect, follow-up 2 × 2 mixed-designs ANOVAs were conducted to identify where interaction would reside along the levels of the independent variable.

Patients were classified into three different categories based on the percentage changes in BMD: “BMD decreased,” “stable,” or “increased.” The criteria for classification were determined according to the least significant change (LSC) of our center's DXA equipment, which was equal to 3.047% at the LS, 3.324% at the TH, and 4.98% at the FN. Subjects whose changes exceeded ± LSC were classified as “BMD increased” or “decreased,” respectively. We included in the model the same covariates as the above-mentioned analyses (treatment, baseline Z-score, baseline ALP and 25OHD).

Two-sided *p*-values of 0.05 or less were considered statistically significant. Data were analyzed using SPSS software, Version 22 (IBM, Inc., Chicago, IL, USA).

## Results

### Patient Characteristics

Twenty-three KTRs receiving treatment with denosumab were enrolled, as well as twenty-three age and sex-matched denosumab-untreated KTRs. The CONSORT flowchart relative to the present study is reported in Supplementary Fig. 1. Of the four excluded individuals of the denosumab group, three were excluded because of incomplete DXA data and one due to the loss in follow-up (moved to a different healthcare facility). The timeframe separating the baseline and follow-up DXA scan was (mean, standard deviation) 49 (3) months (range 42–55 months) for the denosumab group and 49 (4) months (range 41–55 months) for the untreated group (F(1, 44) = 0.14, *p* = 0.71 between groups).

The baseline characteristics of the two groups are reported in Table 1; the distribution of the baseline CKD stages is depicted in Supplementary Fig. 2; none of the patients enrolled presented an eGFR < 30 mL/min/1.73 m^2^. All patients were in stable treatment with a calcineurin inhibitor, mycophenolic acid, and glucocorticoids (5 mg daily of prednisolone equivalent). As expected, the baseline BMD and T-scores at all three sites were significantly lower in the denosumab group, the baseline Z-scores were significantly lower at the LS and TH in the denosumab group, while no significant differences were observed for the other parameters.

**Table 1:** Baseline characteristics of the two groups

	Denosumab (*N* = 23)	Controls (*N* = 23)	*p*-value
Sex (M:F)	10:13	10:13	Ns
Age (years)	61.5 (7.2)	61.4 (9.0)	Ns
Post-menopausal women	9	10	Ns
BMI (kg/m^2^)	23.1 (1.8)	24.3 (4.3)	Ns
T2DM	3 (15%)	3 (15%)	Ns
History of previous haemodialysis	14 (61%)	17 (74%)	Ns
Years from transplant to baseline DXA; median [IQR]	4 [1.5;10]	5 [2;11]	Ns
Previous history of fragility fractures	7	4	Ns
Chronic GCs	23 (100%)	23 (100%)	/
Treatment with calcimimetics	4 (17%)	6 (26%)	Ns
Treatment with active vitamin D compounds	6 (26%)	7 (30%)	Ns
Treatment with active vitamin D analogues	0	0	/
25OHD (nmol/L)	48.6 (26.7)	57.2 (37.3)	0.067
Subjects with 25OHD < 30 nmol/L	6 (26%)	5 (21%)	Ns
Serum corrected calcium (mg/dL)	9.6 (0.5)	9.5 (0.7)	Ns
ALP (U/L)	83.4 (43.5)	77.8 (29.1)	Ns
PTH (pmol/L)	13.0 (10.1)	18.9 (14.8)	Ns
Serum creatinine (mg/dL)	1.31 (0.45)	1.27 (0.43)	Ns
LS T-score	− 2.70 (1.20)	− 1.42 (1.70)	0.006
TH T-score	− 2.35 (0.77)	− 1.40 (1.16)	0.014
FN T-score	− 2.58 (0.52)	− 1.98 (1.04)	0.002
LS Z-score	− 1.62 (1.31)	− 0.60 (1.50)	0.019
TH Z-score	− 1.43 (0.53)	− 0.61 (0.96)	0.001
FN Z-score	− 2.58 (0.51)	− 1.95 (1.02)	0.085
Number of osteoporotic subjects*	22 (95%)	9 (39%)	< 0.001
Ten-year probability of fracture (FRAX **®**); median [IQR]	16 [10.4–21.1]	10 [6.3–14.5]	0.02

Data are expressed as mean (SD) or absolute number (percentage). *defined as having a T-score <  − 2.5 at least one of the three sites. Square brackets show the reference range of normality

*M* males, *F* females, *BMI* body mass index, *T2DM* type 2 diabetes mellitus, *DXA* dual-energy X-rays absorptiometry, *GCs* glucocorticoids, *SD* standard deviation, *IRQ* interquartile range, *LS* lumbar spine, *TH* total hip, *FN* femoral neck, *FRAX* ® Fracture Risk Assessment Tool ®, *Ns* non-significant, *25OHD* 25-hydroxy vitamin D, *ALP* alkaline phosphatase, *PTH* parathyroid hormone

### Primary Endpoints

**Fig. 1:**
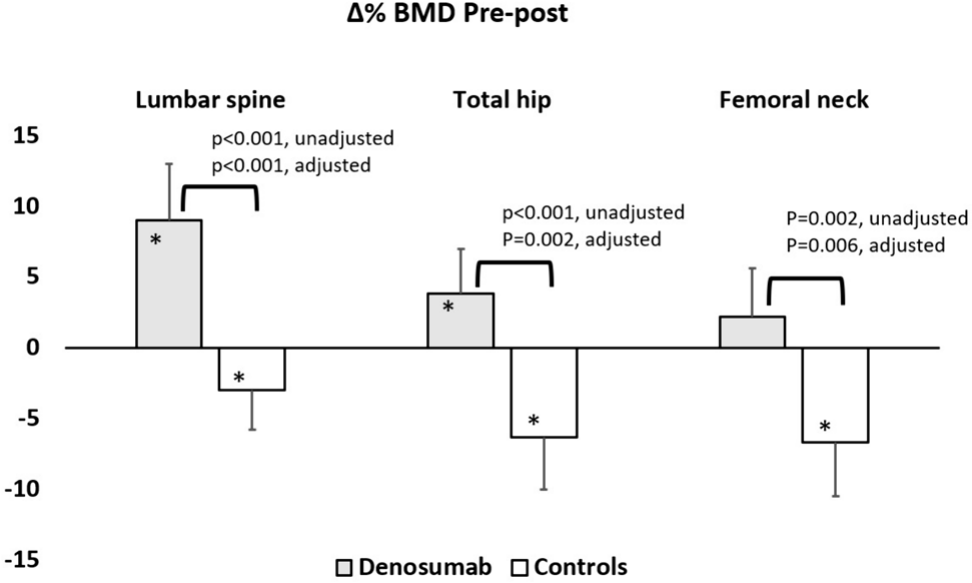
Percentage BMD changes after four years of observation of the denosumab and control groups. Horizontal square brackets and the p-values show the between groups differences, unadjusted and after adjustment for respective baseline Z-score, PTH and 25(OH)D. Error bars show 95% confidence intervals. *p < 0.05 vs baseline

The BMD percent changes for both groups after 4 years of observation are depicted in Fig. 1. The treated group showed a significant increase from baseline in BMD at the LS (9.0 ± 10.7%, F(1, 22) = 22.1, *p* < 0.001 vs baseline) and TH (3.8 ± 7.9%, F(1, 22) = 4.7, *p* = 0.041 vs baseline), and a not significant increase at the FN (2.3 ± 8.3%, F(1, 22) = 1.47, *p* = 0.238 vs baseline). The untreated group showed a significant decrease at all sites (− 3.0 ± 7%, F(1, 22) = 4.69 *p* = 0.041 vs baseline at the LS; − 6.3 ± 9.2%, F(1, 22) = 11.1, *p* = 0.003 vs baseline at the TH; − 6.7 ± 9.3%, F(1, 22) = 11.35, *p* = 0.003 vs baseline at the FN, respectively).

The between groups differences in percent BMD changes were statistically significant at all sites: 12.1% 95% CI [6.7;17.5], (F(1, 22) = 20.6, *p* < 0.001 at the LS, 10.1% 95% CI [4.9;15.4], F(1,22) = 15.3, *p* < 0.001 at the TH and 9.0% 95% CI [3.6;14.4]; F(1,22) = 11.4, *p* = 0.002 at the FN, respectively between groups).

After adjustment for baseline Z-scores, PTH and 25OHD serum levels, the differences remained significant at all sites.

The results of the ordinal logistic regression revealed significantly higher odds of being classified with a better outcome at all three sites (OR 36.7 [7.15–280.93], 12.4 [3.177–57.56], 3.7 [1.092–13.63] at LS, TH, and FN, respectively—see Supplementary Fig. 3 and Supplementary Table 1).

### Secondary Endpoints

**Fig. 2:**
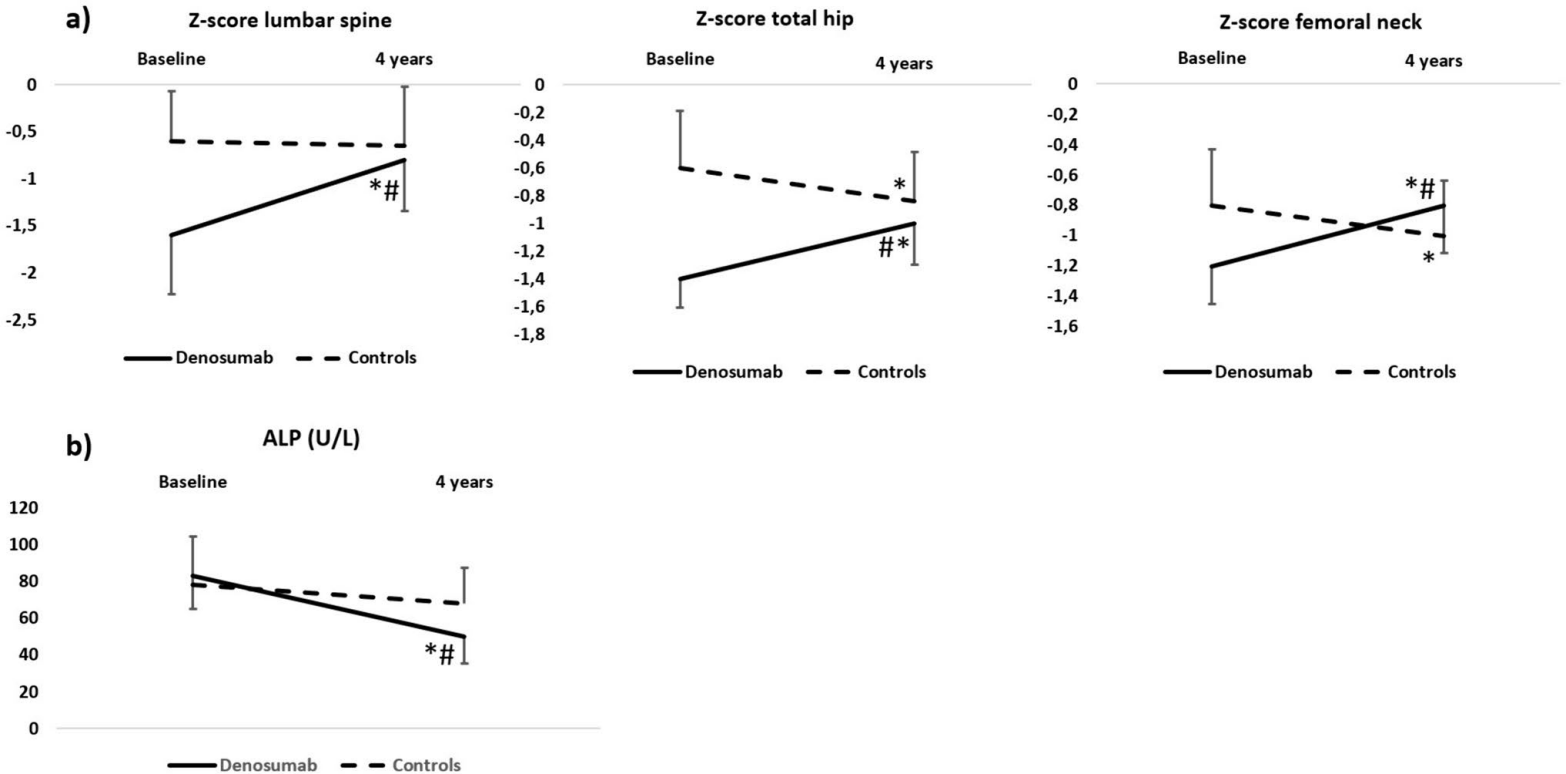
BMD changes expressed as Z-scores after four years of observation. Error bars show 95% confidence intervals. *p < 0.05 vs baseline. #*p* < 0.05 vs controls

The changes in the Z-score at all sites are depicted in Fig. 2, panel a. The Z-scores at all sites significantly increased in the treated group, while they decreased at the femoral neck and total hip in the untreated group. The between-group differences were significant at all sites.

The ALP serum levels significantly decreased from baseline only in the denosumab group (Fig. 2, panel b), with a significant between-group difference (F(1, 44) = 4.92, *p* = 0.032). After adjustment for baseline PTH and 25OHD, the between-group changes remained significant (F(1, 42) = 4.2, *p* = 0.046). The PTH serum levels remained stable over time with no statistically significant differences between the two groups (data not shown), while serum 25OHD increased to 67.4 ± 24.5 nmol/L in the denosumab group (F(1, 22) = 9.93, *p* = 0.005 vs baseline) and to 75.1 ± 26.3 nmol/L in the untreated group (F(1, 22) = 1.62, *p* = 0.21 vs baseline) without a statistically significant between-group difference (F(1, 44) = 0.91, *p* = 0.34). No significant differences over time in serum creatinine were documented for both groups (within and between groups). Over the 4 years, one patient in the denosumab group experienced a fragility fracture (a vertebral fracture). Three patients in the untreated group (two ribs fractures for the first patient, a wrist fracture for the second both for low-energy traumas; two small bones of the foot for the third one, which were classified as stress fractures). All twenty-three denosumab patients continued the treatment without treatment-related adverse events. When follow-up corrected serum calcium levels were compared to baseline, only serum calcium at month six in the untreated group showed a significant difference (*p* = 0.017). In addition, a significant difference in the corrected serum calcium changes was observed between groups (time * treatment interaction effect; F(3, 132) = 3.5, *p* = 0.017) (Supplementary Fig. 4). Follow-up analyses confirmed a significant baseline to month six time * treatment interaction effect (F(1, 44) = 12.7, *p* = 0.001), and baseline to month twenty-four (F(1, 44) = 6.9, *p* = 0.011), while no statistically significant interaction effect was found between month six and twelve and month twelve and twenty-four. During the observation period, five patients in the denosumab group and six patients in the untreated group experienced incident serious infections. No cases of moderate or severe hypocalcaemia (serum calcium corrected for albumin < 8 mg/dL) or acute transplantation rejection were observed during the clinical follow-up in any of the two groups.

## Discussion

We hypothesized that denosumab would be effective in preserving or increasing BMD in KTRs in routine clinical practice, even when there is a significant time gap between transplantation and the start of treatment.

Our study investigated the long-term changes in BMD in KTRs receiving denosumab for 4 years and compared them to the natural history of a matched untreated KTR cohort in a real-life scenario. The denosumab group exhibited significant increases in BMD from baseline at the LS and TH, whereas the untreated cohort experienced significant BMD loss at all sites. Although the data from the literature are limited, our findings are in line with those of previous reports [16–18] and corroborate the effectiveness of denosumab in increasing BMD in this special population.

In addition, the present study adds significant insights on the topic; for instance, the POSTOP study, a 12-month open-label randomized controlled trial [17], showed for the first-time the benefits of denosumab treatment in this special population. However, in that trial, the treatment course was started soon after the transplant surgery, a scenario that may not necessarily be generalizable to those patients whose bone health assessment might have been overlooked for several years. The average time gap between the transplant surgery and the first DXA evaluation in our study was almost 8 years and nevertheless denosumab showed significant BMD gains over the 4 years of follow-up. Furthermore, the treatment period of the POSTOP trial was limited to twelve months, a timeframe in which the untreated group BMD appeared to be stable.

In the present study, at the 4-year mark, a notable difference in the respective trajectories of the two groups becomes evident when considering the Z-score values. As is well-established, the Z-score represents the number of standard deviations that distinguish the patient's data from the mean of a healthy population of the same age, thereby accounting for the natural decline in BMD associated with advancing age over time. A significant decrease from the baseline of the Z-score marks a BMD decline inappropriate for aging and therefore emphasizes the decline in skeletal health of these patients, a well-documented multifactorial phenomenon [19]. Our study suggests that, in KTRs, denosumab may be not only able to stop this trend but even reverse it, as shown by the converging and intersecting lines of the Z-scores depicted in Fig. 2. As a consequence, at the end of the follow-up, several patients of the treated cohort reached or even exceeded the BMD of the untreated individuals.

In a recent retrospective study, McKee et al. compared the effects on BMD of forty-six patients treated with denosumab to thirty-nine receiving bisphosphonates [18] with 3.4 years of follow-up. With a comparable treatment duration, the Authors observed a mean BMD increase at the LS of roughly 7.5%, in line with ours (9.05%), and an increase at the femoral neck of 4.49% (with a calculated 95% confidence interval [CI] of 1.15 to 7.8%), slightly superior but largely overlapping with ours. Conversely, the overall effect size within the bisphosphonate group was significantly lower and without any BMD gain at the femoral neck.

Interestingly, in our study, the net difference in the BMD changes between the denosumab and untreated groups (12%) are very similar at the LS to those observed after 4 years in the FREEDOM extension trial (with a difference vs. placebo of roughly 12%) [20], and even greater at the femoral neck (8.95% vs. roughly 6% of the FREEDOM). In addition, in long-term non-nephropathic glucocorticoid users, a 2.2% BMD increase at the femoral neck was observed in the second year of treatment [21]. Given these premises, it seems that, apart from the chronic glucocorticoid treatment that nearly all KTRs receive, there may be specific kidney transplant-related factors that impede (or slow down) the effectiveness of RANKL inhibition when assessing its benefits in terms of BMD from baseline. However, when we take into account the natural history of this special population, marked by accelerated bone loss, the overall benefit may indeed be preserved (Fig. 2, panel a).

Finally, when denosumab was used in real-world populations, an increased risk of hypocalcaemia was observed, particularly in those with CKD [22]. However, we did not observe any concerning trend suggesting the development of this complication in our treated cohort, albeit the blood samples were taken as per the clinical schedule and not specifically to investigate the nadir of the serum calcium expected after the administration of denosumab (which is expected one to two weeks after its administration) [23].

The present data corroborate the effectiveness of denosumab in increasing BMD at all sites, without significant signals for safety concerns, especially in terms of the risk of hypocalcaemia. The use of denosumab in this patient population is supported by the fact that, unlike bisphosphonates, this molecule does not interfere with kidney function [24], and it has been associated with greater BMD increases also in cases of corticosteroid-induced osteoporosis [25].

Differently from bisphosphonates, denosumab can be associated with an unwanted rebound effect in the case of unsupervised discontinuation [26], a phenomenon also described in KTRs [27], albeit such events might not be so common, even in special populations [28].

The risk of this rebound makes it advisable to initiate bisphosphonate therapy after denosumab cessation [29]. Future studies should focus on developing strategies to mitigate the risk of a rebound effect, especially in those patients who cannot receive treatment with bisphosphonates.

As expected, we observed a decrease in ALP, a commonly adopted surrogate marker of bone turnover, especially in CKD patients. Interestingly, we did not observe significant changes in the levels of serum PTH, probably due to the achievement of adequate levels of 25OHD serum levels and calcium intake. The same measure could also help explain the absence of any episode of significant hypocalcaemia, even though a statistically significant interaction effect for treatment was observed. However, the magnitude of this effect was limited and plateaued at month six. In our opinion, this remark further emphasizes the importance of correct calcium and vitamin D supplementation in these subjects [30].

This study has several limitations. The retrospective design, even after adjusting for the variables of interest included, cannot account for all confounders or uncontrollable exposures. Of note, the two samples differed significantly in baseline BMD. Although we adjusted our analysis for this potential confounder, without a randomized design, we cannot entirely exclude a confounding effect. Furthermore, the limited sample size and single-center nature restrict its generalizability and preclude the drawing of strong conclusions regarding incident fractures and adverse events.

In conclusion, our study showed the benefit of 4 years of denosumab treatment in a real-life setting of KTRs. BMD gains were significant at the lumbar and hip sites, while the untreated population experienced BMD losses at all sites, especially relevant at the total hip and neck.

### Supplementary Information

Below is the link to the electronic supplementary material.

Supplementary file1 (DOCX 310 KB)

## Data Availability

The data underlying this article will be shared on reasonable request to the corresponding author.
