# Supplementary material for: Long-Term Bone Mineral Density Changes in Kidney Transplant Recipients Treated with Denosumab: A Retrospective Study with Nonequivalent Control Group
Source: Calcif Tissue Int. 2024 May 10;115(1):23–30. doi: 10.1007/s00223-024-01218-z (PMC11153264; doi:10.1007/s00223-024-01218-z)
Supplement: Supplementary file 1 — Supplementary file1 (DOCX 310 KB) [file 223_2024_1218_MOESM1_ESM.docx]

**Supplementary table and figures**


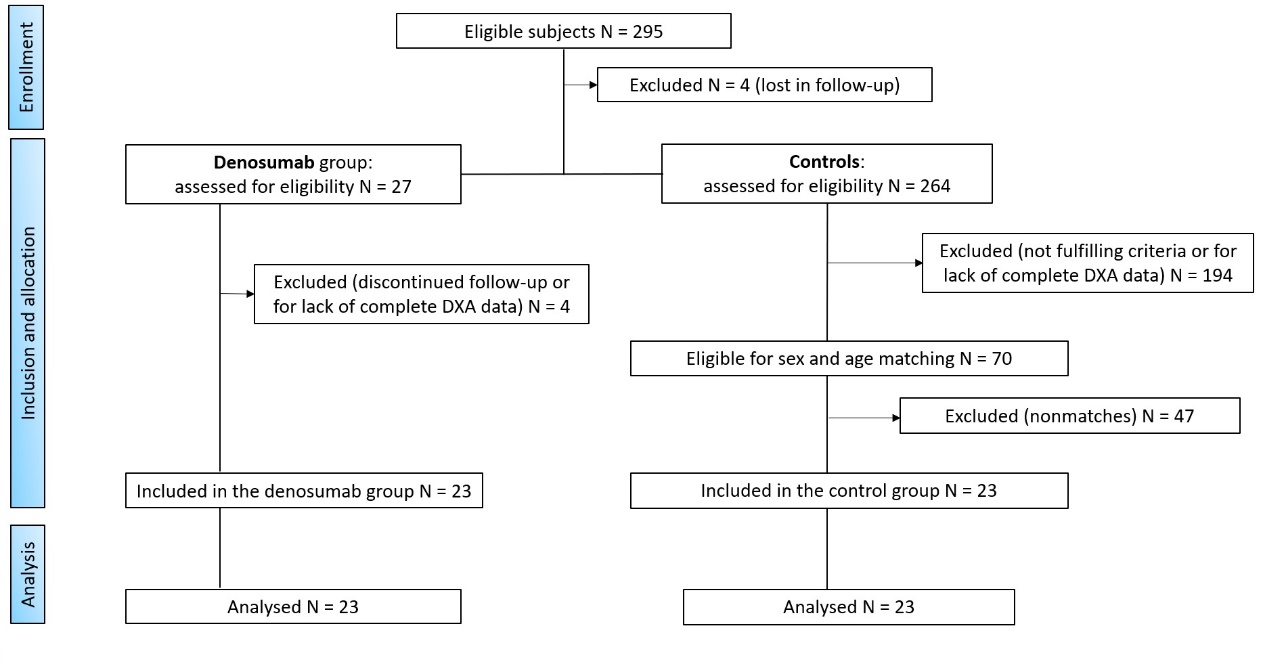


**Supplementary Figure 1**: the CONSORT flowchart of the present study.


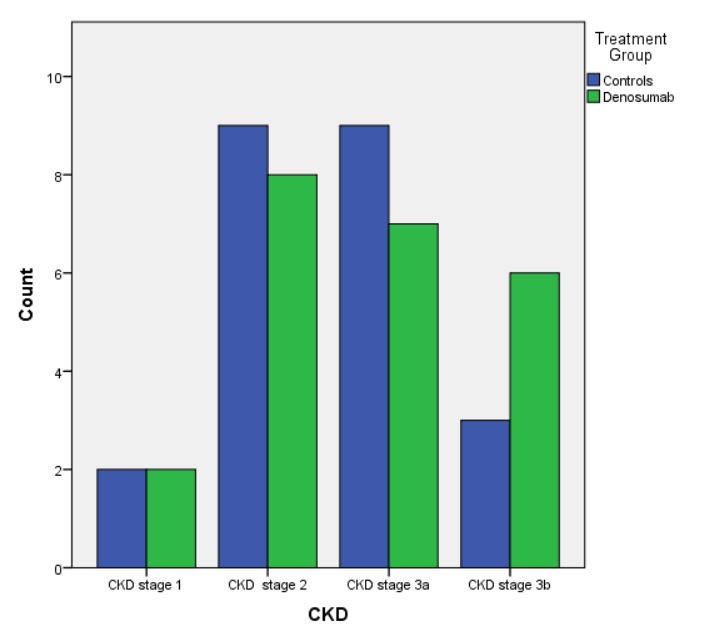


**Supplementary Figure 2**: the distribution of the baseline chronic kidney disease (CKD) stages classified according to eGFR of the denosumab and control groups. CKD stage was defined according to the respective baseline eGFR.


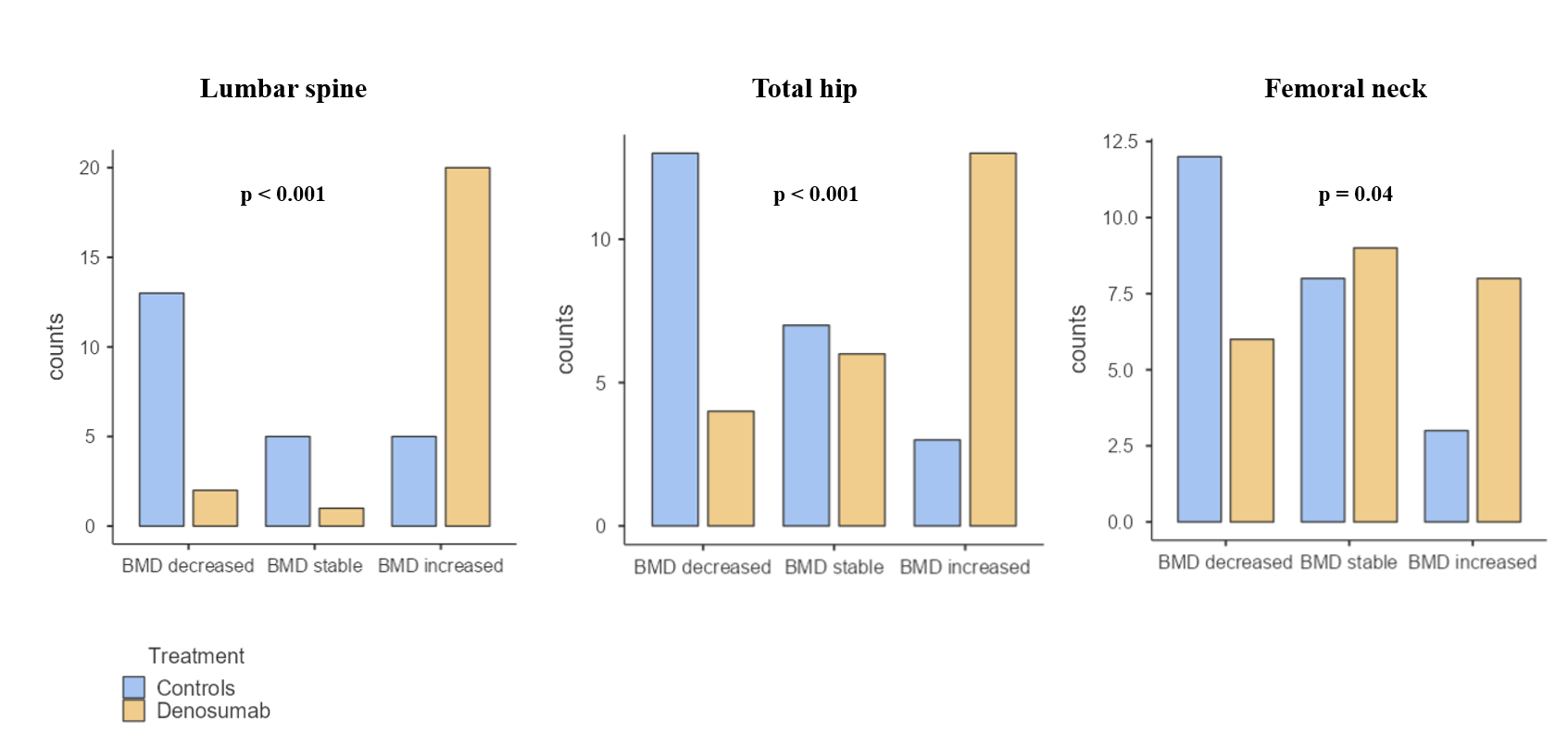


**Supplementary Figure 3**: the distribution (absolute counts) of the subjects classified as “BMD increased”, “stable” or “decreased”. P-values refers to the ordinal logistic regression analysis (see text).


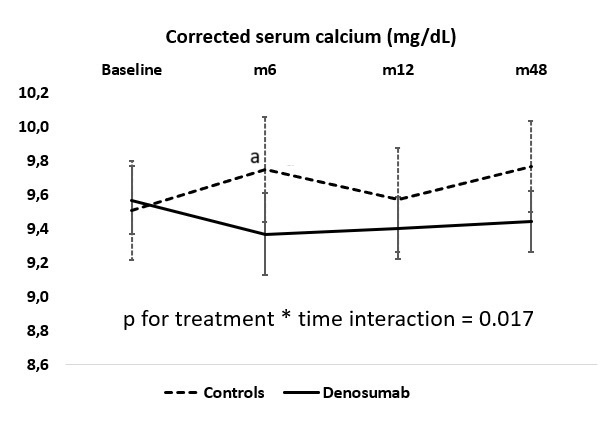


**Supplementary Figure 4**: mean levels of serum calcium corrected for albumin of the two groups. A significant treatment * time interaction effect was observed between the two groups. Error bars show 95% confidence intervals. a p<0.05 vs baseline.

| **Lumbar spine BMD** (order: BMD decreased – stable – increased) | | |
| --- | --- | --- |
| *McFadden R2: 0.27* | **OR [95%CI]** | **p-value** |
| **Treatment-denosumab (controls: ref)** | 36.7 [7.15-280.93] | <0.001 |
| Baseline LS Z-Score | 1.130 [0.699-1.87] | 0.620 |
| Baseline ALP | 0.989 [0.968-1.01] | 0.243 |
| Baseline 25OHD | 1.011 [0.989-1.04] | 0.354 |
| **Total hip BMD** (order: BMD decreased – stable – increased) | | |
| *McFadden R2: 0.14* | **OR [95%CI]** | **p-value** |
| **Treatment-denosumab (controls: ref)** | 12.4 [3.177-57.56] | <0.001 |
| Baseline LS Z-Score | 1.20 [0.795-1.87] | 0.389 |
| Baseline ALP | 1 [0.982-1.02] | 0.979 |
| Baseline 25OHD | 1.013 [0.994-1.03] | 0.185 |
| **Femoral neck BMD** (order: BMD decreased – stable – increased) | | |
| *McFadden R2: 0.063* | **OR [95%CI]** | **p-value** |
| **Treatment-denosumab (controls: ref)** | 3.7 [1.092-13.63] | 0.040 |
| Baseline LS Z-Score | 1.189 [0.796-1.82] | 0.410 |
| Baseline ALP | 1.007 [0.991-1.03] | 0.394 |
| Baseline 25OHD | 0.997 [0.978-1.02] | 0.764 |

**Supplementary table 1**: ordinal logistic regression for BMD percentage changes at lumbar spine, total hip or femoral neck. Change status classified according to the least significant change thresholds.
